# Lesion activity assessment of early caries using dye-enhanced quantitative light-induced fluorescence

**DOI:** 10.1038/s41598-022-15862-8

**Published:** 2022-07-13

**Authors:** Seok-Woo Park, Si-Mook Kang, Hyung-Suk Lee, Sang-Kyeom Kim, Eun-Song Lee, Bo-Ra Kim, Elbert de Josselin de Jong, Baek-Il Kim

**Affiliations:** 1 Department of Preventive Dentistry & Public Oral Health, BK21 FOUR Project, Yonsei University College of Dentistry, Seoul, 03722 Republic of Korea; 2 R&D Center, AIOBIO Co., Ltd., Seoul, 06241 Republic of Korea; 3 Department of Dental Hygiene, Namseoul University, Cheonan-si, 31020 Republic of Korea; 4 Department of Health Service Research, University of Liverpool, Liverpool, L69 7ZX UK; 5 Inspektor Research System BV, 1402CG Bussum, The Netherlands; 6 Innovation Research and Support Center for Dental Science, Yonsei University Dental Hospital, Seoul, 03722 Republic of Korea

**Keywords:** Medical research, Optics and photonics

## Abstract

We aimed to determine whether dye-enhanced quantitative light-induced fluorescence (DEQLF), wherein porous structure of caries lesions is stained with a fluorescent dye, could quantitatively distinguish between active and inactive caries. A total of 126 bovine specimens were prepared to artificially simulate caries activity. Active caries were demineralized with 1% carbopol solution for 3 (A*3*), 5 (A*5*), and 10 days (A*10*). For inactive caries, half specimens in each group were remineralized with 2% NaF and reallocated into three groups (I*3*, I*5*, and I*10*, respectively). Wet specimens were dried with compressed air for 10 s and then dyed with 100-µM sodium fluorescein for 10 s. Fluorescence images of speicmens were captured with a QLF-digital 2 + Biluminator. Fluorescence intensity (ΔG) was measured in fluorescence images of dyed specimens. ΔG between active and inactive groups was compared using independent *t*-test, and ΔG among active groups (or inactive groups) were compared using ANOVA (α = 0.05). ΔG in the active groups was 33.7–59.0 higher than that in the inactive groups (*P* < 0.001). Except between I3 and I5, there was significant differences in ΔG according to the demineralization period (*P* < 0.001). DEQLF might be used to evaluate early caries activity, and longitudinally monitor changes in lesion activity.

## Introduction

Imbalances in the dynamic process of tooth demineralization and remineralization lead to the development of early caries lesions with an intact surface^1–4^. When demineralization predominates, the surface porosity of caries lesions increases, and when remineralization predominates, surface porosity decreases^5,6^. Caries lesions with progressing demineralization are defined as active, whereas those with arrested demineralization are defined as inactive^7,8^. Active caries lesions continue to increase in surface porosity due to the progression of demineralization. In contrast, in inactive caries lesions where remineralization predominates, the surface porosity decreases, which either stops the progression of the lesion or promotes recovery to sound tissue^5,9^. Therefore, surface porosity may be an important factor in determining lesion activity in early caries.

Quantitative methods for measuring the surface porosity of early caries lesions have been used to objectively determine lesion activity. Micro-computed tomography (micro-CT) and transverse microradiography (TMR) could be used to evaluate the lesion porosity by measuring the mineral content via X-ray absorption profiles. Previous studies have reported that the surface layer of an active lesion is less porous than that of an inactive lesion^10,11^. However, micro-CT and TMR require extracted or sliced teeth as specimens; therefore, the two methods are limited to use in laboratory environment. Additionally, the radiation emitted by micro-CT and TMR may be harmful to the human body and are therefore not suitable for clinical studies. Thus, it is necessary to develop a non-invasive method of evaluating surface porosity and determining lesion activity in early caries for clinical use.

The visual-tactile method has been widely used for lesion activity assessment. It qualitatively measures the roughness and reflection of the lesion surface, which are properties related to surface porosity^7,9,12^. For example, when the surface porosity increases, the surface layer of the caries lesion becomes irregular, resulting in a rough texture, dull-whitish color, and loss of luster^13–15^. In previous studies quantitatively evaluating roughness and reflection, no difference was observed in roughness between active and inactive lesions. However, qualitative studies have shown that the reflection of active lesions is significantly higher than that of inactive lesions^13^. Therefore, reflection may be used to determine lesion activity. However, when measuring reflection in the clinic, the teeth are first dried with compressed air. Thus, the visual-tactile method can be an unreliable, subjective method of assessing lesion activity, as variations can occur according to the skill and ability of the examiner while observing the dull-whitish-colored lesion.

In dentistry, there have been efforts to objectively and quantitatively evaluate caries lesions using optical technologies. Among them, quantitative light-induced fluorescence (QLF) technology uses blue visible light of 405-nm wavelength to detect oral diseases (e.g., dental caries, tooth wear, tooth fissure, and pathogenic dental plaque)^16–23^. When an early caries lesion is irradiated with blue visible light, it emits a relatively dark autofluorescence compared to sound enamel, owing to light scattering by the porous structure of the surface layer^24–26^. When early caries lesions are dried with compressed air, the active lesions show significantly darker autofluorescence than the inactive lesions as shown in an in vitro study^14^. However, there is no statistically significant difference in the fluorescence intensity between active and inactive lesions in the in vivo study^27^. Because the changes in the fluorescence intensity by air-drying was too small to reflect the difference of caries lesion surface characteristics, therefore, there is a need to reveal the difference of the caries lesion surface characteristics in the fluorescence images in a dental clinic.

A fluorescent dye may be used by clinicians to detect and monitor invisible early caries lesions, as it can penetrate the porous structure of caries lesions by capillary action^28^. Fluorescein sodium (molecular weight of 376.27) is one such fluorescent dye that emits yellow-green autofluorescence under UV light. It is only produced synthetically and does not form an ionic bond with enamel^28,29^. Therefore, it can be used to fill the porous structure of caries lesions with bright autofluorescence without any physical and chemical reactions. Dye-enhanced QLF (DEQLF) is a method of imaging and quantifying fluorescence by applying a fluorescent dye to a caries lesion^30–32^. In a previous study evaluating artificial root caries lesions using DEQLF, there was better penetration of fluorescein sodium into demineralized lesions, resulting in a brighter autofluorescence^33^. In addition, one pilot study showed the potential of fluorescein sodium in distinguishing the activity of natural early caries^34^. However, the severity of natural caries can be a confounding factor.

Thus, the possibility of using fluorescein sodium to differentiate the lesion activity of early caries at the same lesion severity needs to be confirmed. Therefore, the aim of this study was to determine whether DEQLF could be used to distinguish between active and inactive lesions. We hypothesized that active and inactive caries lesions can be quantitatively and intuitively distinguished using the DEQLF.

## Results

**Figure 1:**
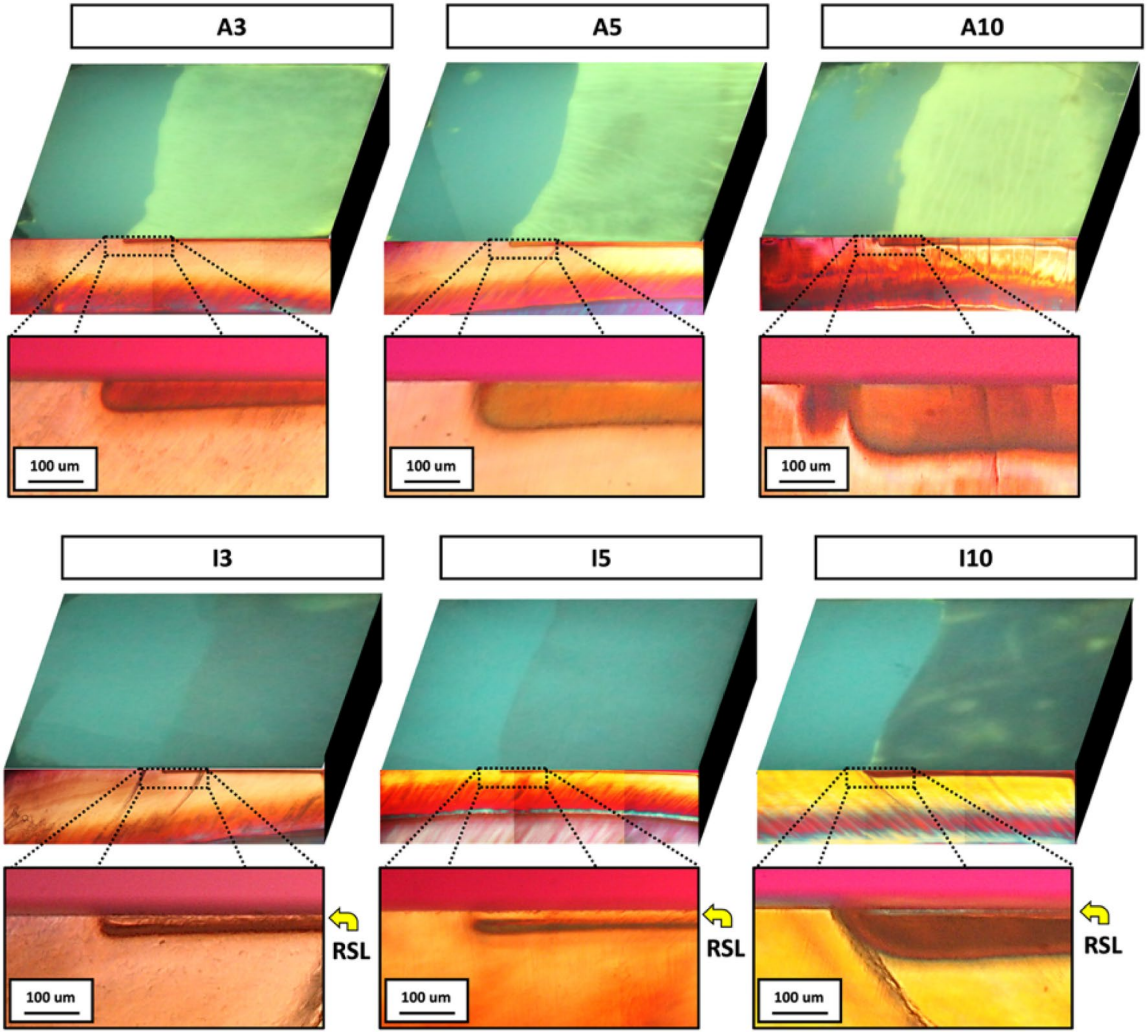
Representative images of the dyed specimens between the active (A*X*) and the inactive (I*X*) groups. Fluorescence images (top view) and the respective polarized-light micrographs (cross-sectioned view, 100 × of magnification). A*X* indicates active lesions that demineralize for *X* days; I*X* indicates inactive lesions that re-mineralize for 10 days after demineralization for *X* days. RSL indicates surface layer of remineralized caries lesion.

The histological and fluorescence images of the representative specimens are presented in Fig. 1. Lesion depth (Ld) (mean ± standard deviation; unit: µm) showed no significant differences between the active and inactive groups in A*3* (38.7 ± 8.2) and I*3* (43.7 ± 10.6), and in A*5* (56.6 ± 13.2) and I*5* (45.7 ± 16.5). However, A*10* (116.6 ± 11.4) was significantly deeper than that in I*10* (76.2 ± 14.1, *P* < 0.001). Yellow arrows in the histological image indicate the surface layer of the remineralized caries lesions. In the caries lesions in I*3* and I*5*, the surface layer showed the same color as that of sound enamel, whileas in the caries lesion of I*10*, the color of the surface layer was different from that of sound enamel. In the fluorescence images of the specimens in all active groups, the caries lesions emitted the yellow-green fluorescence of the fluorescent dye. Among the inactive groups, yellow-green fluorescence was not observed in the caries lesions in I*3* and I*5*, whereas the caries lesion in I*10* emitted the yellow-green fluorescence of the fluorescent dye, point-by-point.

The ΔG of the specimens were compared between the active and the inactive groups (Table 1). Among ΔG of the dried specimens, all active groups (negative values) were significantly 11.2–20.2 lower than all inactive groups (positive values, *P* < 0.001). In contrast, among ΔG of the dyed specimens, all active groups (positive values) were significantly 33.7–59.0 higher than all inactive groups (positive values, *P* < 0.001).

**Table 1:** Comparison of ΔG of the dried and dyed specimens between active (A*X*) and inactive (I*X*) groups in different demineralization periods.

Demineralization period (day)	Dried specimen			Dyed specimen		
	A*X*	I*X*	*P* value	A*X*	I*X*	*P* value
3	− 21.1 (6.6)	− 9.9 (5.0)	< 0.001	24.0 (12.6)	− 9.7 (4.8)	< 0.001
5	− 27.4 (5.3)	− 9.0 (6.8)	< 0.001	38.9 (11.3)	− 10.2 (5.5)	< 0.001
10	− 38.9 (7.3)	− 18.7 (8.7)	< 0.001	46.4 (16.2)	− 12.6 (9.8)	< 0.001

Mean (standard deviation).

ΔG is defined as the brightness of caries lesion compared to that of sound enamel (unit: %).

A*X* indicates active lesions that demineralize for *X* days; I*X* indicates inactive lesions that re-mineralize for 10 days after demineralization for *X* days.

The ΔG of the specimens were compared among lesions of various severity (demineralization periods) in all the active groups. In the ΔG of the dried specimens, A*5* and A*10* were significantly 1.6 times and 1.9 times lower than A*3* (*P* < 0.001), and A*10* was significantly 1.4 times lower than A*5* (*P* < 0.001). In contrast, in the ΔG of the dyed specimens, A*5* and A*10* were significantly 1.3 times and 1.8 times higher than A*3* (*P* < 0.001), and A*10* was significantly 1.2 times higher than A*5* (*P* < 0.001).

The ΔG of the specimens were also compared among lesions of various severity in all the inactive groups. In the ΔG of the dried specimens, I*5* showed no significant difference with I*3*, and I*10* was significantly 1.9 times and 2.0 times lower than I*3* and I*5*, respectively (*P* < 0.001). In contrast, in the ΔG of the dyed specimens, there was no significant difference among the different lesion severities.

**Figure 2:**
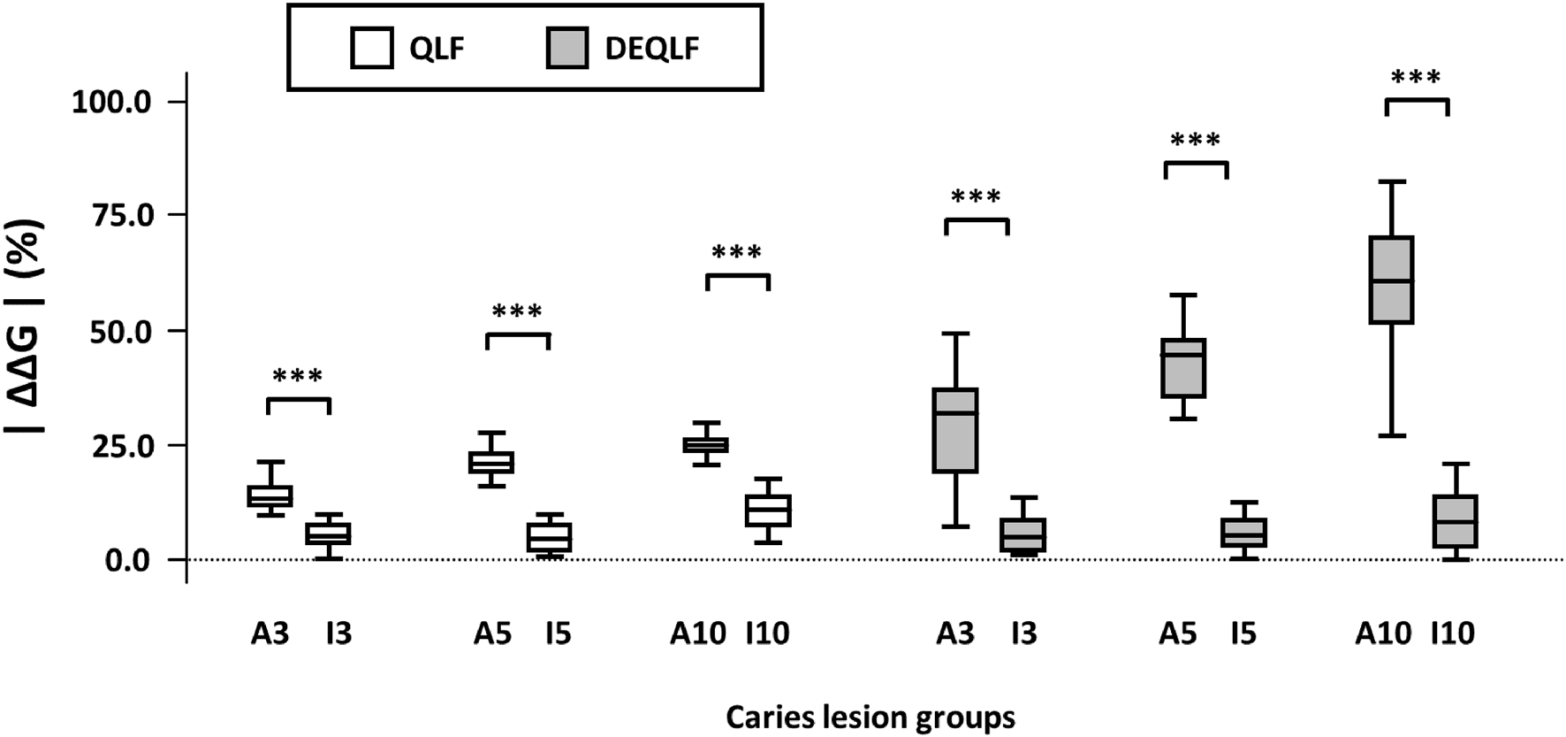
Box plots of |ΔΔG| of the specimens between active (A*X*) and inactive (I*X*) groups. |ΔΔG| is defined as the absolute value of the difference in ΔG between the specimen subjected to QLF and DEQLF. A*X* indicates active lesions that demineralize for *X* days; I*X* indicates inactive lesions that re-mineralize for 10 days after demineralization for *X* days. *P *value was calculated by independent *t* test (α = 0.05). ***P < 0.001, **P < 0.01, *P < 0.05.

When the specimens were subjected to DEQLF, the change in fluorescence (|ΔΔG|) in active groups was 5.2–7.4 times higher than that in inactive groups (*P* < 0.001, Fig. 2). On the contrary, when the specimens were subjected to QLF, the change in fluorescence (|ΔΔG|) in active groups was 2.2–4.3 times higher than that in inactive groups.

The |ΔΔG| of the specimens were compared among lesions of various severity in all the active groups. In the |ΔΔG| of the dried specimens, A*5* and A*10* were significantly 1.5 times and 1.7 times lower than A*3* (*P* < 0.001), and A*10* was significantly 1.1 times lower than A*5* (*P* < 0.001). In contrast, in the |ΔΔG| of the dyed specimens, A*5* and A*10* were significantly 1.5 times and 2.0 times higher than A*3* (*P* < 0.001), and A*10* was significantly 1.3 times higher than A*5* (*P* < 0.001).

Likewise, the |ΔΔG| of the specimens were also compared among lesions of various severity in all the inactive groups. In the |ΔΔG| of the dried specimens, I*5* showed no significant difference with I*3*, and I*10* was significantly 1.9 times and 2.1 times lower than I*3* and I*5*, respectively (*P* < 0.001). Also, in the |ΔΔG| of the dyed specimens, I*5* was not significantly different from I*3*, and I*10* was significantly 3.5 times and 3.2 times higher than I*3* and I*5*, respectively (*P* < 0.001).

## Discussion

In this study, we investigated whether DEQLF could differentiate between active and inactive lesions in early caries. On visual observation of the fluorescence images of specimens subjected to DEQLF, active caries lesions emitted brighter autofluorescence than inactive caries lesions. DEQLF was used to distinguish whether the early caries lesions were active or inactive by observing autofluorescence with the naked eye in caries lesions. By calculating the ΔG, the lesion activity of early caries could be quantitatively evaluated.

Among the lesions subjected to DEQLF, the autofluorescence of the active caries was brighter than that of the sound enamel and showed a positive ΔG, whereas the autofluorescence of the inactive caries lesions was darker than that of the sound enamel and showed a negative ΔG (Table 1). This finding is similar to the results of previous studies, in which active caries lesions showed brighter fluorescence than inactive lesions when DEQLF was performed on artificial root caries lesions^33^. These results can be explained by the fact that the porosity of the surface of a caries lesion increases proportionally as the number and diameter of microchannels in the surface layer increase^35,36^. Likewise, in the histological evaluation in this study, we confirmed that the active caries lesions have the surface layer that color was different from that of sound enamel (Fig. 2). However, because the surface porosity was not measured in this study, future research is needed to evaluate the relationship between the ΔG values and surface porosity.

QLF and DEQLF can help differentiate between sound enamel and early caries. In all the studied groups, sound enamel and caries lesions showed different fluorescence patterns in the fluorescent images. A previous study also confirmed that sound enamel and artificial caries lesions (including the shallowest lesion, demineralized for only 2 h) showed different fluorescence patterns of the fluorescent dye pyrromethene 556^32^. However, it is still questionable whether the fluorescein sodium can indicate caries lesion severity. In this study, the fluorescence parameters of specimens (conducted by QLF or DEQLF) were not different in inactive groups, while there was a significant difference in active groups among different demineralization periods. It was also confirmed that there was no significant difference in the fluorescence parameter of the pyrromethene 556 among different caries lesion depths in the previous study^32^. The inactive lesions used in this study were designed with varied surface porosities by remineralizing the surfaces using 2% sodium fluoride. Within relatively shallow lesions (3 days and 5 days of demineralization period), the surface layer seemed well remineralized in the histological images. Therefore, no difference in ΔG and lΔΔGl between I*3* and I*5* means that the fluorescent parameter changes could be indicative of surface changes in the caries lesions only.

In the fluorescence image of the specimens subjected to DEQLF, the inactive caries lesions of I*10* emitted partially bright autofluorescence of the fluorescent dye, while the inactive caries lesions of I*3* and I*5* emitted dark autofluorescence (Fig. 1). However, in the histological images, the inactive caries lesions of I*3* and I*5* showed a similar lesion surface layer to that of sound enamel; the inactive caries lesions of I*10* showed a similar lesion surface layer to that of demineralized lesions. When the caries lesions were treated with high-concentration fluoride (such as 2% sodium fluoride), the remineralization of the surface layer differed according to the lesion severity^37^. Therefore, it is considered that the caries lesions of I*10* had a “surface layer of less remineralized” due to the high concentration of fluoride at a relatively deep lesion depth. Considering this finding, DEQLF could be used to detect inactive lesions in which the lesion surface is less remineralized, by observing the autofluorescence of a fluorescent dye that is partially emitted within the lesion. DEQLF may also be used to evaluate the extent to which the lesion has remineralized.

DEQLF can potentially help clinicians to assess lesion activity. Among the dried specimens in this study, both the active and inactive groups emitted darker fluorescence than sound enamel (Table 1). In previous studies that used QLF to observe early caries lesions during drying, both active and inactive lesions emitted dark fluorescence resulting from the evaporation of water from the porous structure of the lesion, as the refractive index changed from water (1.33) to air (1.00)^14,27,34^. However, it is difficult for clinicians to facilitate evaporation of such a small amount of water as observed in the early caries lesions, and to assess lesion activity by visually observing or quantitatively measuring the result of evaporation (change in the refractive index of the lesion). However, in this study, it was possible to clearly differentiate between active and inactive lesions using DEQLF because of the bright fluorescence of the active lesions and dark fluorescence of the inactive lesions (Fig. 1). Therefore, these results show that DEQLF might have potential as a lesion activity assessment, to visualize and quantify the porous structure of early caries lesions.

Fluorescein sodium was used as the fluorescent dye in this study. Previous studies have reported that porphyrin derivatives are mainly used as fluorescent dyes for caries lesion assessment with laser fluorescence (LF)^38–42^. However, the porphyrin derivatives are not suitable as a fluorescent dye in DEQLF, because they already exist in natural caries lesions as bacterial metabolites^20,43–46^. Consequently, when performing DEQLF on natural caries lesions with porphyrin derivatives, it is impossible to distinguish the red fluorescence between the fluorescent dye and the bacterial metabolite. In contrast, since fluorescein sodium emits yellow-green autofluorescence, it can be easily distinguished from the red autofluorescence of porphyrin derivatives in caries lesions. Therefore, fluorescein sodium is useful as a fluorescent dye in DEQLF, to evaluate the lesion activity of natural caries. However, since this study used artificial lesions to simulate active and inactive lesions in the laboratory, it was not possible to reflect all features (dental plaque, dental calculus, and anatomical morphology) of natural caries lesions.

This is the first study to distinguish between active and inactive lesions in artificial caries using DEQLF. We confirmed that there was a difference in the autofluorescence emitted from the active and inactive lesions of artificial caries using fluorescence dye in DEQLF. Active lesions emitted brighter yellow-green autofluorescence than sound enamel, whereas inactive caries lesions emitted darker autofluorescence than sound enamel. DEQLF may be a supportive modality for clinicians in the intuitive and quantitative assessment of lesion activity in early caries, based on different fluorescence patterns and surface porosity.

Except between the relatively shallow lesions (groups I*3* and I*5*), DEQLF is useful for quantitatively distinguishing active and inactive lesions based on differences in autofluorescence. Therefore, DEQLF might be an objective assessment of lesion activity. The method could become a useful tool for assessing lesion activity in a single observation and for longitudinal monitoring of lesion activity in clinical practice. Additionally, DEQLF is a simple procedure; therefore, even dentists with little experience in evaluating lesion activity of early caries can easily use this technology in clinical settings.

## Methods

### Specimen preparation

**Figure 3:**
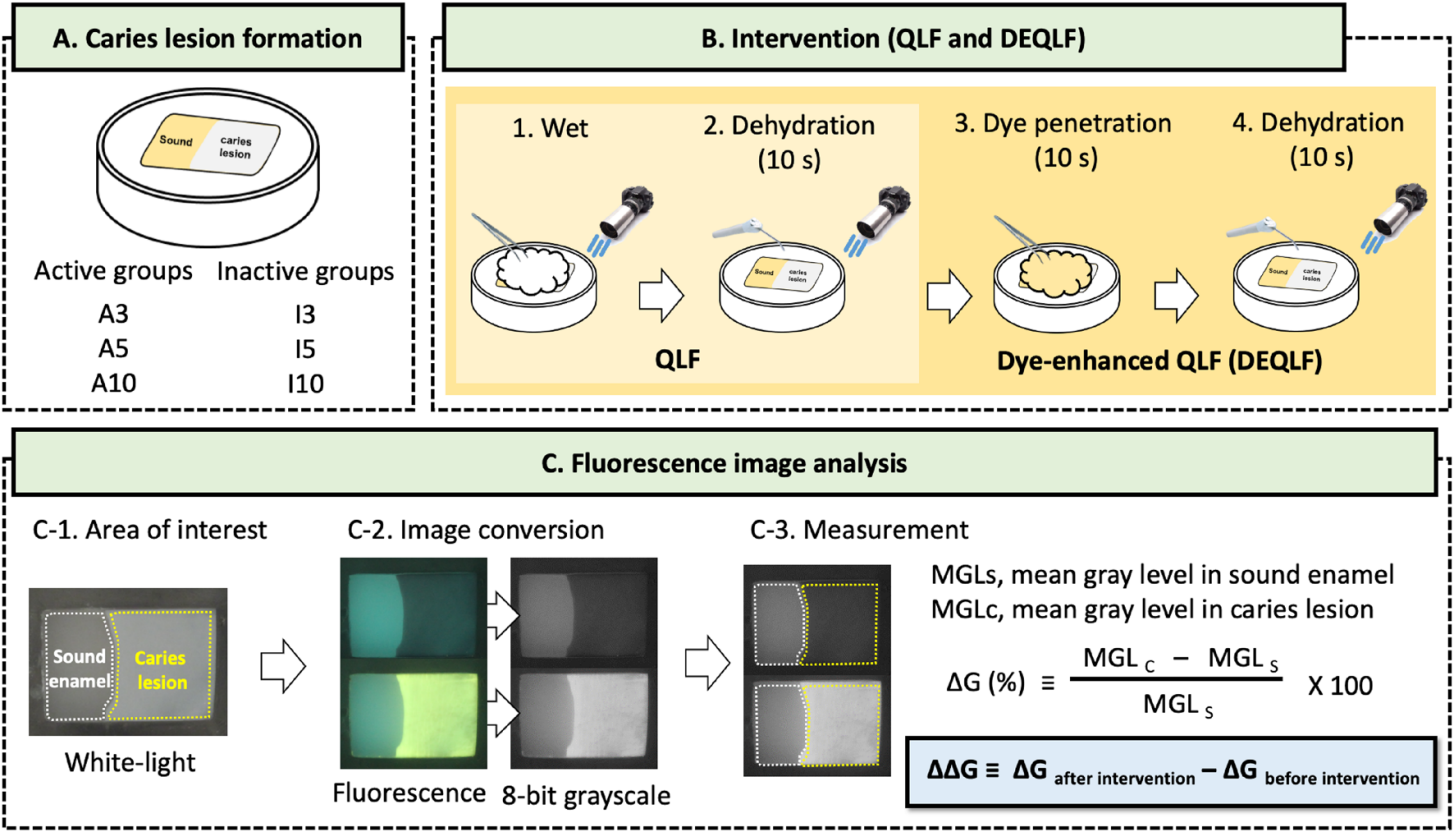
Schematic diagram of this in vitro study. (**A**) specimen allocation, (**B**) the process of Quantitative Light-induced Fluorescence (QLF) and dye-enhanced QLF (DEQLF), (**C**) the process of fluorescence image analysis for calculating ΔG (fluorescence intensity of caries lesions) and ΔΔG (changes in ΔG by the intervention).

Bovine maxillary jaws were purchased in the slaughterhouse for preparing bovine tooth specimen. A total of 63 teeth with no dental cracks, dental caries, dental fluorosis, or enamel hypoplasia on the labial surfaces were obtained from the jaws using a low-speed diamond disk (NTI-KAHLA GmbH, Kahla, Germany). Based on the results (average ± standard deviation; 5.9 ± 8.7 of the active group and 0.4 ± 0.7 of the inactive group) of the previous study, 0.8 power and 0.05 type I error (α) were expected, and the minimum sample size of 21 samples per group was established^34^. The teeth were then cut to make 126 slabs (4 × 3 × 3 mm) using the low-speed diamond disk (NTI-KAHLA GmbH). With the labial surface facing outward, the slab was embedded in acrylic molds (20 × 12 × 7 mm) with 9-mm diameter holes using self-cured resin (Ortho-Jet, Lang Dental Manufacturing, Wheeling, Illinois, USA; Fig. 3A). The surface of the specimen was polished using a water-cooled polishing machine (RB 209 Minipol, R&B Inc., Daejeon) with 400-, 800-, and 1200-grit abrasive papers (SiC sandpaper, R&B Inc., Daejeon, South Korea). Half of the surface was covered with an acid-resistant and non-fluorescent transparent varnish to protect the sound enamel.

### Caries lesion formation

In this study, caries lesions were artificially formed to simulate active and inactive lesions. Demineralized and remineralized lesions were used to reproduce the active and inactive lesions, respectively.

The specimens were equally distributed among three active groups (A*X*, where *X* indicates the demineralization period; Fig. 3A). The demineralizing gel contained 0.1 M lactic acid gel (pH 4.8) with 1% carbopol (Carbopol^®^ ETD 2050 polymer, Noveon Inc., Wickliffe, Ohio, USA), and was 50% saturated with hydroxyapatite (calcium phosphate tribasic Sigma, St. Louis, MO, USA)^47^. The specimens were immersed in 40 mL of the gel at 37 °C during the demineralization period in each group (3, 5, and 10 days, respectively). After the specified durations, the specimens were removed from the gel and washed with distilled water.

Half of the specimens from each group (21 per group) were reassigned to three inactive groups (I*X*, where *X* indicates the demineralization period; Fig. 3A). The remineralization procedure consisted of four steps^36^. First, the specimens were immersed in 3 mL of 2% sodium fluoride solution for 4 min. Second, the solution remaining on the surface of the specimen was washed with distilled water. Third, the specimens were immersed in artificial saliva (0.22% mucin, 30 mM KCl, 10 mM KH_2_PO_4_, 13 mM NaCl, and 3 mM CaCl_2_, final pH 6.8; refilled daily) for the rest of the day. Finally, the solution remaining on the surface of the specimen was rinsed with distilled water. The entire remineralization procedure was repeated for 10 days. After the remineralization process was complete, the acid-resistant varnish covering the surface was removed using acetone. Except for one specimen, in which the nail varnish was removed in the I*10* group during the demineralization process, 125 specimens were used until the end of this study. The specimens were placed in a dark container and stored in a refrigerator at 4 °C until performing DEQLF.

### QLF and Dye-enhanced QLF (DEQLF)

QLF is a process comprising hydration and subsequent dehydration (Fig. 3B). First, cotton pellets saturated with distilled water were placed on the surface to hydrate the specimens for 60 s. The specimens were then dehydrated for 10 s using compressed air^14,35^. DEQLF is a process comprising QLF, dye penetration, additional dehydration (Fig. 3B). First, cotton pellets saturated with the fluorescent dye, 100 μM fluorescein sodium salt (Sigma-Aldrich, St. Louis, MO, USA) in 50% ethanol solution, were placed on the surface of the dried specimens for 10 s^48^. According to a previous study, 100 μM sodium fluorescein emitted sufficiently bright fluorescence in the caries lesion, and it was confirmed that the caries lesion activity could be evaluated by applying sodium fluorescein for 10 s. The fluorescent dye residue on the surface of the specimen was removed for 10 s using distilled water. The specimens were then additionally dehydrated for 10 s using compressed air.

White light and fluorescent images of the wet, dried, and dyed specimens were captured using QLF-digital 2 + Biluminator (QLF-D, Inspector Research Systems BV, Amsterdam, The Netherlands). Imaging conditions were as follows: white light image (shutter speed 1/90 s, aperture value 13.0, ISO speed 1600) and fluorescent image (shutter speed 1/30 s, aperture value 13.0, ISO speed 1600).

### Image analysis

All images were analysed using an image analysis software (Image-Pro PLUS 6.0, Media Cybernetics, Rockville, MD, USA). The fluorescence images of all specimens were converted into 8-bit grayscale images using the “convert to” function (Fig. 3C). The area of interest (AOI) of sound enamel and caries lesions was drawn along the margins of the sound enamel and caries lesions, respectively. The mean gray levels in the AOIs of sound enamel (Gs) and caries lesions (Gc) measured using an 8-bit grayscale image. The fluorescence intensity (ΔG) of the caries lesion with respect to sound enamel was calculated using the following formula: ${\Delta G} \left( \% \right) = \frac{{\left( {{\text{Gc}} - {\text{Gs}}} \right)}}{{{\text{Gs}}}} \times 100$. A positive ΔG indicates that the caries lesion emits brighter fluorescence compared to the sound enamel. In contrast, a negative ΔG means that the caries lesion emits darker fluorescence compared to the sound enamel. To measure the fluorescence change in the caries lesion according to the intervention (QLF and DEQLF), ΔΔG (ΔG _after intervention_ – ΔG _before intervention_) was calculated before and after the intervention, respectively. To be able to compare the difference in G, the absolute ΔΔG (lΔΔGl) was used as the fluorescence parameter.

### Histological analysis

The straight line containing the point with the highest gray level parallel to the major axis of the specimen was set as the AOI. The specimen was cut vertically twice using a microtome (TechCut 4™, Allied High Tech Products, Compton, CA, USA) for the acquisition of the sectioned tooth, including the AOI. It was ground using 800-grit abrasive papers until the thickness of the sectioned tooth was 150 µm. The sectioned tooth was located between a cover glass and a glass slide with distilled water as the medium. Histological images (× 400 magnification) of the sectioned teeth were obtained using a polarizing microscope. In the histological image, the Ld of early caries was calculated using an image analysis program (Image Pro PLUS 6.0, Media Cybernetics).

### Statistical analysis

All statistical analyses were performed using SPSS version 21.0 (IBM Corp., Armonk, NY, USA). The difference in ΔG, and |ΔΔG| of the specimens among lesions of various severity (demineralization periods) was evaluated using one-way ANOVA followed by tukey post hoc (α = 0.05). The difference in Ld, ΔG, and |ΔΔG| of the specimens between the active and inactive groups was evaluated using an independent *t*-test (α = 0.05).

## References

[1] Takahashi N, Nyvad B (2008). Caries ecology revisited: Microbial dynamics and the caries process. Caries Res..

[2] Featherstone JDB (2004). The continuum of dental caries—evidence for a dynamic disease process. J. Dent. Res..

[3] Arends J, Christoffersen J (1986). Invited review article: The nature of early caries lesions in enamel. J. Dent. Res..

[4] Hariri I (2013). Estimation of the enamel and dentin mineral content from the refractive index. Caries Res..

[5] Holmen L, Thylstrup A, Artun J (1987). Surface changes during the arrest of active enamel carious lesions in vivo: A scanning electron microscope study. Acta Odontol. Scand..

[6] Artun J, Thylstrup A (1986). Clinical and scanning electron microscopic study of surface changes of incipient caries lesions after debonding. Scand. J. Dent. Res..

[7] Nyvad B, Fejerskov O (1997). Assessing the stage of caries lesion activity on the basis of clinical and microbiological examination. Commun. Dent. Oral. Epidemiol..

[8] Pitts, N. B. *Detection, Assessment, Diagnosis and Monitoring of Caries* (ed. Lussi, A. A. & Whitford, G.M.) 63–90 (Ekstrand, K.R., Zero, D.T., Martignon, S., Pitts, N.B., 2009).

[9] Thylstrup A, Bruun C, Holmen L (1994). In vivo caries models-mechanisms for caries initiation and arrestment. Adv. Dent. Res..

[10] Deyhle H, White SN, Bunk O, Beckmann F, Müller B (2014). Nanostructure of carious tooth enamel lesion. Acta Biomater..

[11] Cochrane NJ (2012). An X-ray microtomographic study of natural white-spot enamel lesions. J. Dent. Res..

[12] Ekstrand KR, Martignon S, Ricketts DJN, Qvist V (2007). Detection and activity assessment of primary coronal caries lesions: A methodologic study. Oper. Dent..

[13] Ando M, Shaikh S, Eckert G (2018). Determination of caries lesion activity: Reflection and roughness for characterization of caries progression. Oper. Dent..

[14] Ando M (2018). Objective and quantitative assessment of caries lesion activity. J. Dent..

[15] Neuhaus KW, Nyvad B, Lussi A, Jaruszewski L (2011). Evaluation of perpendicular reflection intensity for assessment of caries lesion activity/inactivity. Caries Res..

[16] Jung E-H (2018). Development of a fluorescence-image scoring system for assessing noncavitated occlusal caries. Photodiagn. Photodyn. Ther..

[17] Ko H-Y, Kang S-M, Kim H-E, Kwon H-K, Kim B-I (2015). Validation of quantitative light-induced fluorescence-digital (QLF-D) for the detection of approximal caries in vitro. J. Dent..

[18] Kim E-S (2017). A new screening method to detect proximal dental caries using fluorescence imaging. Photodiagn. Photodyn. Ther..

[19] Jun M-K (2016). Detection and analysis of enamel cracks by quantitative light-induced fluorescence technology. J. Endod..

[20] Lee H-S (2018). Caries detection and quantification around stained pits and fissures in occlusal tooth surfaces with fluorescence. J. Biomed. Opt..

[21] Kim S-K, Jung H-I, Kim B-I (2020). Detection of dentin-exposed occlusal/incisal tooth wear using quantitative light-induced fluorescence technology. J. Dent..

[22] Park S-W (2020). Clinical assessment of an automated fluorescent plaque index scoring with quantitative light-induced fluorescence. Photodiagn. Photodyn. Ther..

[23] Lee E-S, de Josselin de Jong Elbert, Jung H-I, Kim B-I (2018). Red fluorescence of dental biofilm as an indicator for assessing the efficacy of antimicrobials. J Biomed Opt..

[24] Amaechi B, Higham SM (2002). Quantitative light-induced fluorescence: A potential tool for general dental assessment. J. Biomed. Opt..

[25] Tranaeus S, Heinrich-Weltzien R, Kühnisch J, Stösser L, Angmar-Månsson B (2001). Potential applications and limitations of quantitative light-induced fluorescence in dentistry. Med. Laser Appl..

[26] de Josselin de Jong Elbert (1995). A new method for in vivo quantification of changes in initial enamel caries with laser fluorescence. Caries Res..

[27] Ando M, Ferreira-Zandoná A, Eckert G, Zero D, Stookey G (2017). Pilot clinical study to assess caries lesion activity using quantitative light-induced fluorescence during dehydration. J. Biomed. Opt..

[28] Romanchuk KG (1982). Fluorescein. Physicochemical factors affecting its fluorescence. Surv. Ophthalmol..

[29] van der Veen MH, Tsuda H, Arends J, ten Bosch JJ (1996). Evaluation of sodium fluorescein for quantitative diagnosis of root caries. J. Dent. Res..

[30] Eggertsson H (1999). Detection of early interproximal caries in vitro using laser fluorescence, dye–enhanced laser fluorescence and direct visual examination. Caries Res..

[31] Ferreira-Zandoná AG, Analoui M, Schemehorn BR, Eckert GJ, Stookey GK (1998). Laser fluorescence detection of demineralization in artificial occlusal fissures. Caries Res..

[32] Ando M (1997). Relative ability of laser fluorescence techniques to quantitate early mineral loss in vitro. Caries Res..

[33] Pretty IA, Ingram GS, Agalamanyi EA, Edgar WM, Higham SM (2003). The use of fluorescein-enhanced quantitative light-induced fluorescence to monitor de- and re-mineralization of in vitro root caries. J. Oral. Rehabil..

[34] Park, S.-W. *et al.* Evaluation of penetration patterns of fluorescein related to caries activity. https://iadr.abstractarchives.com/abstract/19iags-3182190/evaluation-of-penetration-patterns-of-fluorescein-related-to-caries-activity (2019).

[35] Ando M, Stookey GK, Zero DT (2006). Ability of quantitative light-induced fluorescence (QLF) to assess the activity of white spot lesions during dehydration. Am. J. Dent..

[36] Braga MM, Mendes FM, Ekstrand KR (2010). Detection activity assessment and diagnosis of dental caries lesions. Dent. Clin. N. Am..

[37] Kim H-E, Kwon H-K, Kim B-I (2013). Recovery percentage of remineralization according to severity of early caries. Am. J Dent..

[38] NouhzadehMalekshah S, Fekrazad R, Bargrizan M, Kalhori KAM (2019). Evaluation of laser fluorescence in combination with photosensitizers for detection of demineralized lesions. Photodiagn. Photodyn. Ther..

[39] Mendes F, de Oliveira E, de Faria D, Nicolau J (2006). Ability of laser fluorescence device associated with fluorescent dyes in detecting and quantifying early smooth surface caries lesions. J. Biomed. Opt..

[40] Lee E-S, Kang S-M, Ko H-Y, Kwon H-K, Kim B-I (2013). Association between the cariogenicity of a dental microcosm biofilm and its red fluorescence detected by quantitative light-induced fluorescence-digital (QLF-D). J. Dent..

[41] Kim Y-S, Lee E-S, Kwon H-K, Kim B-I (2014). Monitoring the maturation process of a dental microcosm biofilm using the quantitative light-induced fluorescence digital (QLF-D). J. Dent..

[42] Kim B-R, Kang S-M, de Josselin de Jong Elbert, Kwon H-K, Kim B-I (2019). In vitro red fluorescence as an indicator of caries lesion activity. Oper. Dent..

[43] Dietel W, Pottier R, Pfister W, Schleier P, Zinner K (2007). 5-Aminolaevulinic acid (ALA) induced formation of different fluorescent porphyrins: A study of the biosynthesis of porphyrins by bacteria of the human digestive tract. J. Photochem. Photobiol. B..

[44] Buchalla W (2005). Comparative fluorescence spectroscopy shows differences in noncavitated enamel lesions. Caries Res..

[45] Park S-W (2019). Comparison of fluorescence parameters between three generations of QLF devices for detecting enamel caries in vitro and on smooth surfaces. Photodiagn. Photodyn. Ther..

[46] Lee Y-R, Kim H-E (2020). Red fluorescence threshold for assessing the lesion activity of early caries. Photodiagn. Photodyn. Ther..

[47] White DJ (1987). Use of synthetic polymer gels for artificial carious lesion preparation. Caries Res..

[48] van der Veen MH, ten Bosch JJ (1993). An in vitro evaluation of fluorescein penetration into natural root surface carious lesions. Caries Res..

